# Metabolomic Fingerprint of Heart Failure with Preserved Ejection Fraction

**DOI:** 10.1371/journal.pone.0124844

**Published:** 2015-05-26

**Authors:** Beshay N. Zordoky, Miranda M. Sung, Justin Ezekowitz, Rupasri Mandal, Beomsoo Han, Trent C. Bjorndahl, Souhaila Bouatra, Todd Anderson, Gavin Y. Oudit, David S. Wishart, Jason R. B. Dyck, Alberta HEART

**Affiliations:** 1 Department of Pediatrics, University of Alberta, Edmonton, Alberta, Canada; 2 Cardiovascular Research Center, University of Alberta, Edmonton, Alberta, Canada; 3 Mazankowski Alberta Heart Institute, University of Alberta, Edmonton, Alberta, Canada; 4 Department of Medicine, University of Alberta, Edmonton, Alberta, Canada; 5 Department of Biological Sciences, University of Alberta, Edmonton, Alberta, Canada; 6 The Metabolomics Innovation Centre (TMIC), University of Alberta, Edmonton, Alberta, Canada; 7 Department of Computing Science, University of Alberta, Edmonton, Alberta, Canada; 8 Libin Cardiovascular Institute of Alberta, Department of Cardiac Sciences, University of Calgary, Calgary, Alberta, Canada; Mayo Clinic, UNITED STATES

## Abstract

**Background:**

Heart failure (HF) with preserved ejection fraction (HFpEF) is increasingly recognized as an important clinical entity. Preclinical studies have shown differences in the pathophysiology between HFpEF and HF with reduced ejection fraction (HFrEF). Therefore, we hypothesized that a systematic metabolomic analysis would reveal a novel metabolomic fingerprint of HFpEF that will help understand its pathophysiology and assist in establishing new biomarkers for its diagnosis.

**Methods and Results:**

Ambulatory patients with clinical diagnosis of HFpEF (n = 24), HFrEF (n = 20), and age-matched non-HF controls (n = 38) were selected for metabolomic analysis as part of the Alberta HEART (Heart Failure Etiology and Analysis Research Team) project. 181 serum metabolites were quantified by LC-MS/MS and ^1^H-NMR spectroscopy. Compared to non-HF control, HFpEF patients demonstrated higher serum concentrations of acylcarnitines, carnitine, creatinine, betaine, and amino acids; and lower levels of phosphatidylcholines, lysophosphatidylcholines, and sphingomyelins. Medium and long-chain acylcarnitines and ketone bodies were higher in HFpEF than HFrEF patients. Using logistic regression, two panels of metabolites were identified that can separate HFpEF patients from both non-HF controls and HFrEF patients with area under the receiver operating characteristic (ROC) curves of 0.942 and 0.981, respectively.

**Conclusions:**

The metabolomics approach employed in this study identified a unique metabolomic fingerprint of HFpEF that is distinct from that of HFrEF. This metabolomic fingerprint has been utilized to identify two novel panels of metabolites that can separate HFpEF patients from both non-HF controls and HFrEF patients.

**Clinical Trial Registration:**

ClinicalTrials.gov NCT02052804

## Introduction

Heart failure (HF) affects more than 5 million people in North America, with approximately 825,000 incident cases per year in the United States alone [1]. Although the incidence of HF has declined since the mid-1990s, the prevalence of HF is still increasing due to improved survival after HF diagnosis [2]. Despite the advent of several new medications, devices and multidisciplinary clinics to manage HF, the prognosis of HF remains poor, with an estimated survival rate of 50% within 5 years after initial HF diagnosis [1]. During the initial clinical work-up for patients with confirmed or suspected HF, patients are classified via ejection fraction (EF) into one of two groups: a) HF with reduced ejection fraction (HFrEF) or b) HF with preserved ejection fraction (HFpEF). HFpEF is becoming increasingly recognized as a distinct clinical entity and accounts for 30–40% of all HF cases [3, 4]. HFpEF is broadly defined as a clinical syndrome in which patients present with symptoms and signs of HF, but normal or near normal left ventricular (LV) systolic function, with or without evidence of abnormal diastolic function (e.g. abnormal LV relaxation, filling, diastolic distensibility and diastolic stiffness) [5]. Patients with HFpEF have a similar one-year mortality as patients with HFrEF [6]. Although many therapies exist for HFrEF, no therapy to date has shown any significant reduction in morbidity and mortality for HFpEF [7].

Several pathophysiologic differences between HFpEF and HFrEF have been identified [8]; however, the molecular pathways responsible for these differences are still poorly understood. Metabolomic analysis has been frequently utilized to elucidate new molecular and pathophysiological processes of several cardiovascular diseases and heart failure [9–12]. Accordingly, a unique metabolomic fingerprint of HFpEF patients may enhance our understanding of the molecular/physiological basis of HFpEF, which may lead to the development of new therapies to specifically treat HFpEF.

Current definitions for the diagnosis of HFpEF, including those endorsed by clinical guidelines [7, 13], are neither sensitive nor specific, and lack the diagnostic accuracy needed for clinical use [14]. Although several biomarkers have been utilized to help in HF diagnosis (e.g. B-type Natriuretic Peptide (BNP) and N-terminal pro-BNP (NT-proBNP)), most of these biomarkers do not provide enough utility to distinguish between HFrEF and HFpEF in clinical practice [15]. Since metabolomics is increasingly being utilized to identify new biomarkers in cardiovascular medicine [16], the second objective of this study is to identify novel metabolite biomarkers to distinguish patients with HFpEF from control subjects, and to distinguish between the two clinical syndromes of HFpEF and HFrEF.

## Materials and Methods

### Participants

Ambulatory patients were selected for metabolomic analysis as part of the Alberta HEART (Heart Failure Etiology and Analysis Research Team; www.albertaheartresearch.ca) project [17] (ClinicalTrials.gov registration: NCT02052804). Briefly, Alberta HEART enrolled outpatients with known HF (both HFrEF and HFpEF) and a range of non-HF control subjects spanning normal healthy subjects to patients without HF but with other clinical diseases. Recruitment in the Alberta HEART study began in January 2010 and as of March 31, 2014, 649 patients have been enrolled. Although our study is a cohort study without a clinical trial intervention or randomization of any kind and thus not subject to the ICMJE statement, we chose to register our observational study for the same purposes desired by the ICMJE statement, albeit after patient enrollment had begun. This was to ensure complete transparency of our study and any study that may emanate from its findings.

**Fig 1:**
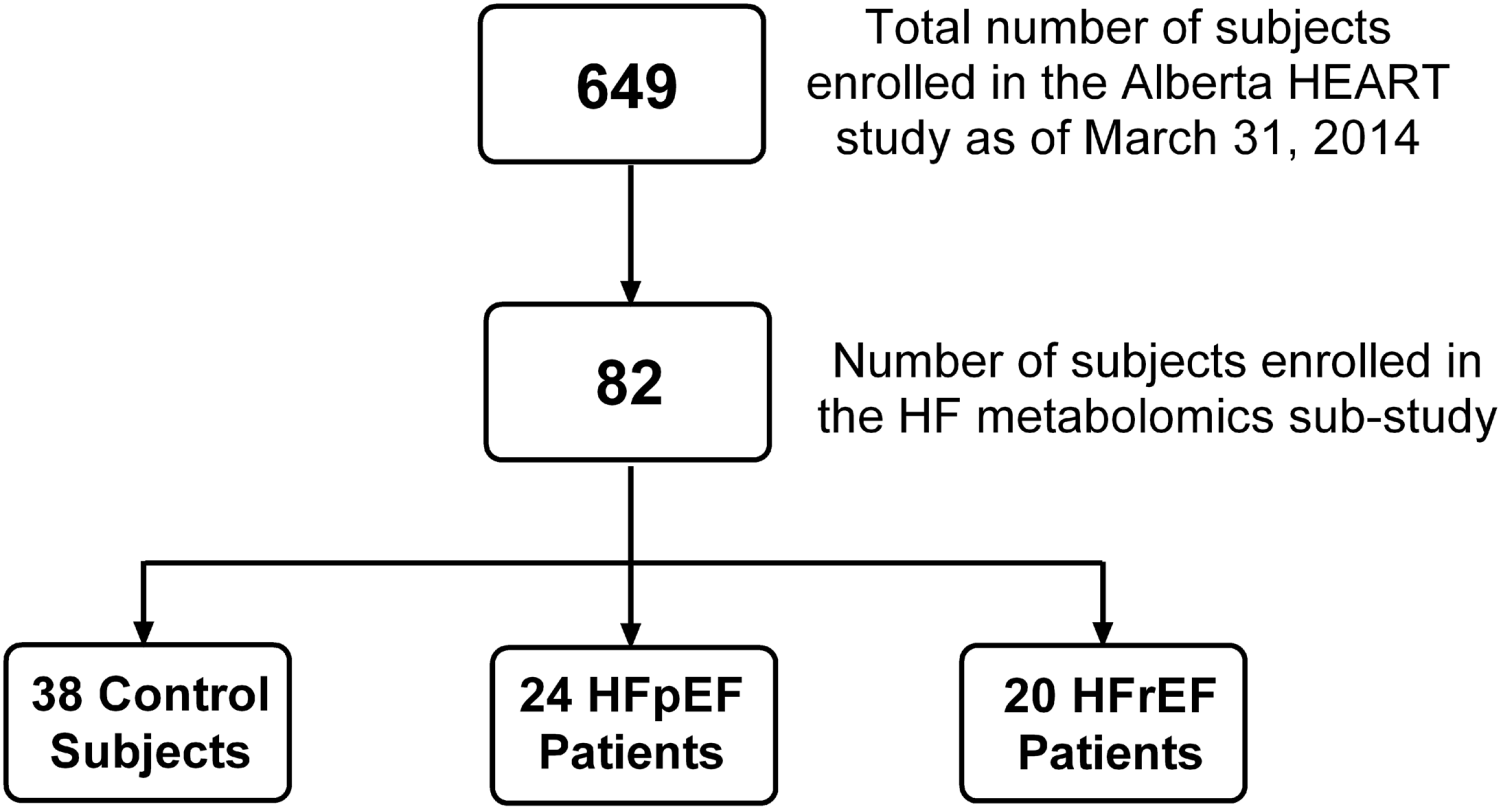
Flow chart representing patient selection in the metabolomics sub-study. 649 patients were enrolled in the Alberta HEART study as of March 31^st^, 2014. Only 82 subjects were included in this metabolomics sub-study. Among these 82 subjects, there were 24 patients with HFpEF, 20 patients with HFrEF, and 38 control subjects without heart failure.

Only 82 subjects were included in this metabolomics sub-study. Among these 82 subjects, there were 24 patients with HFpEF, 20 patients with HFrEF, and 38 control subjects without HF. Participants in this metabolomics sub-study were recruited from January 2010 till March 2013 (Fig 1). In the current study, control subjects are defined as individuals with normal LV function as assessed by echocardiography and without symptoms suggestive of a clinical diagnosis of HF. The control subjects included age- and gender-matched individuals with no evidence of coronary artery disease (CAD), hypertension, diabetes mellitus, organ disease or replacement therapies; no evidence of inflammatory or autoimmune conditions and not on cardiac medications. Additional controls included age- and gender-matched patients with high risk of developing HF but no clinically overt HF; these latter patients are generally asymptomatic (no dyspnea or fatigue) and have no known prior HF or other overt cardiovascular disease. The exclusion criteria included: severe liver disease, end stage renal disease (with GFR < 15 ml/min), active or ongoing malignancy, or cardiac surgery, major surgery, or major cardiovascular event in the past 3 months. All enrolled patients undergo blinded adjudication by cardiologists experienced in adjudication. For the purposes of this study, patients with HF were further adjudicated as HFpEF (n = 24) or HFrEF (n = 20) using a left ventricular ejection fraction (LVEF) of 45% as a cutoff value [5, 13]. Detailed clinical, biomarker and imaging data were collected at the time of enrolment and echocardiograms were performed in 2D, 3D, and contrast modalities and interpreted by cardiologists blinded to the metabolomic analysis. The research protocol used in this study received ethics board approval at the Universities of Alberta and Calgary. Written informed consent was obtained from all subjects.

### Measurement of cardiac peptides

Plasma BNP levels were assessed as previously described [18] using a Biosite Triage reagent pack (Biosite Inc., San Diego, CA, USA), while plasma NT-proBNP analysis was performed with the commercially available immunoassay using the Elecsys 2010 proBNP assay (Roche Diagnostics GmbH, Manheim, Germany) read in an automated Access immunoanalyzer (Beckman-Coulter, Fullerton,CA, USA) at Alberta Health Services Laboratory Services—Edmonton, Alberta.

### Combined direct flow injection and LC-MS/MS compound identification and quantification

We applied a targeted quantitative metabolomic approach to analyze the serum samples using a commercially available metabolomics system (Absolute*IDQ* p180 Kit—BIOCRATES Life Sciences AG, Austria). This kit, in combination with an ABI 4000 Q-Trap (Applied Biosystems/MDS Sciex) mass spectrometer equipped with a reverse-phase HPLC column, can be used for the targeted identification and quantification of up to 180 different endogenous metabolites including amino acids, acylcarnitines, biogenic amines, glycerophospholipids, sphingolipids and sugars. The kit contains reagents for the derivatization and extraction of analytes (for maximal separation), along with the software to support selective mass-spectrometric detection via multiple reaction monitoring (MRM) pairs (for metabolite identification and quantification). Isotope-labeled internal standards and other internal standards are integrated in the kit plate filter to permit absolute metabolite quantification. All the serum samples were analyzed with the AbsoluteIDQ p180 kit using the protocol described in the AbsoluteIDQ user manual.

### Sample preparation and NMR spectroscopy

Serum samples were prepared as described previously [19]. A total of 350 μL of sample was transferred to a micro-cell NMR tube (Shigemi, Inc., Allison Park, PA) for subsequent spectral analysis. All ^1^H-NMR spectra were collected on a 500 MHz Inova NMR spectrometer (Varian Inc. Palo Alto, CA) equipped with a 5 mm HCN Z-gradient pulsed-field gradient (PFG) room-temperature probe as described previously [19].

### Statistical analysis

For demographic and clinical data, continuous data are presented as median ± interquartile range (IQR), while categorical data are presented as raw values and percentages from the total. Continuous variables were compared using a Kruskal-Wallis test, while categorical data were compared through a Chi-square test. In situations where less than 5 observations were available, a Fisher`s Exact test was applied. A p-value < 0.05 was considered significant for all statistical analyses.

For metabolomic data analysis, log-transformation was applied to all quantified metabolites to normalize the concentration distributions. Heat maps were generated with the concentrations of potential candidate metabolites, which were extracted with univariate analysis. It was generated without hierarchical cluster analysis unlike usual structure of heat map. It was simply arranged by grouping similar metabolites together for use in pathway analysis through intuitive pattern discovery. The heat map displays an increase in each metabolite in relative concentration as a red color and a decrease in a metabolite as a blue color. The metabolites are listed at the left side of each row, and the subjects are shown at the bottom of each column. Logistic regression (LR) was performed to find the most parsimonious model to discriminate each case group from the other control groups using the minimum number of metabolites. In order to optimize the metabolite (i.e. variable) selection, a technique called least absolute shrinkage and selection operator (LASSO) was also performed. A receiver operating characteristic (ROC) curve was determined for each LR model. The ROC calculations included bootstrap 95% confidence intervals for the desired model specificity as well as other measures including accuracy and false discovery rates (FDR). In addition, permutation tests (n = 2,000) were performed to validate the statistical significance of each LR model [20]. The p-value of the permutation test was calculated as the proportion of the times that the class separation for a randomly labelled sample was at least as good as the one based on the original data (one-sided p value) [21].

LR models to discriminate HFpEF from HFrEF, or HF from controls were constructed. To assess if a biomarker or a panel of metabolites improved the categorization of a patient, the ROC area-under-the-curve (AUC), net reclassification improvement (NRI) and integrated discrimination improvement (IDI) were calculated using the methods described by Pencina et al. [22]. All of the statistical analyses were carried out using the R statistical software (http://www.r-project.org).

## Results

### Demographics and group differences

**Fig 2:**
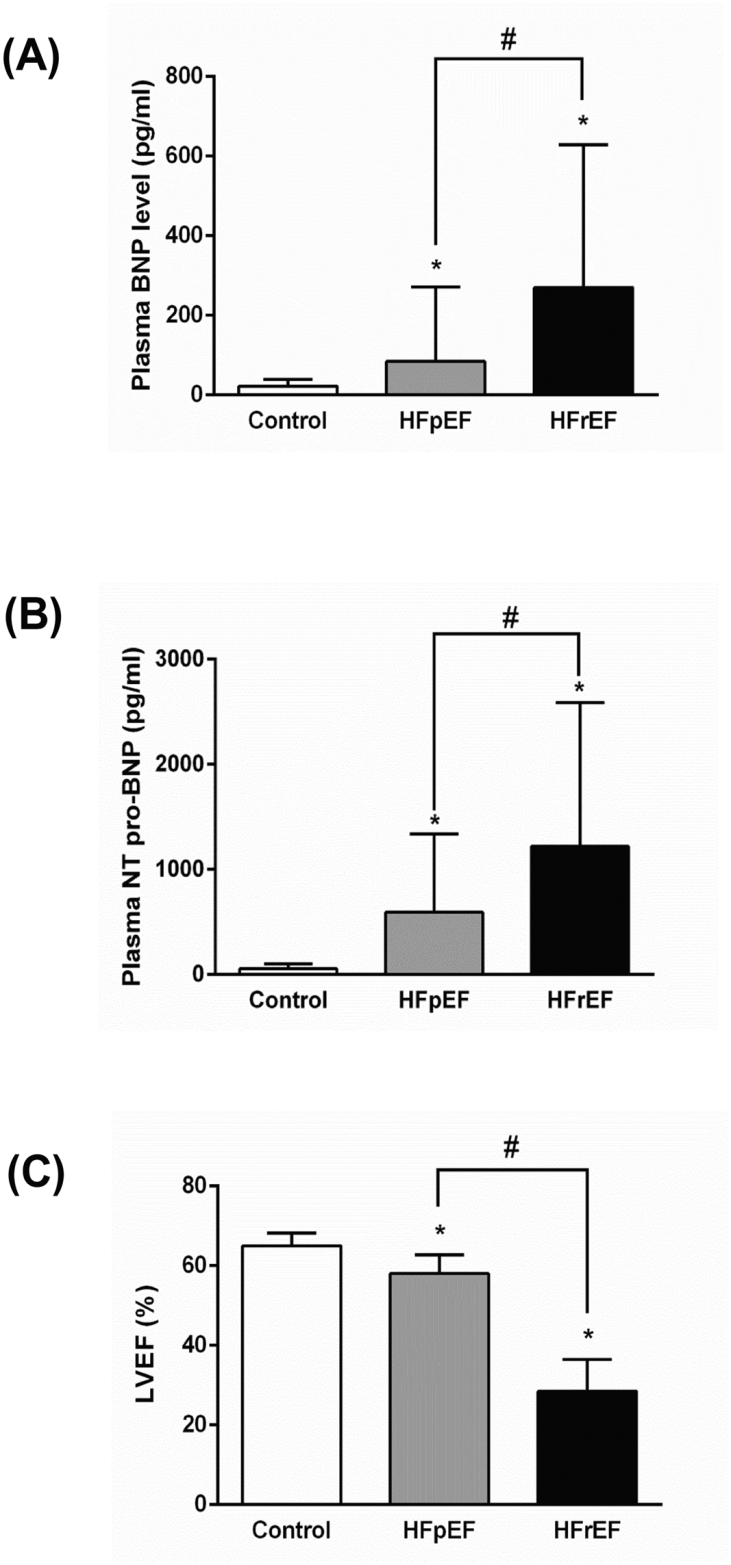
Cardiac peptides and left ventricular ejection fraction (LVEF) in control and HF patients. Ambulatory patients with clinical diagnosis of HFpEF (n = 24), HFrEF (n = 20), and age-matched controls (n = 38) were selected for metabolomics analysis as part of the Alberta HEART (Heart Failure Etiology and Analysis Research Team) project. Plasma BNP and NT-proBNP levels were measured using a Biosite Triage reagent pack and Elecsys 2010 proBNP assay, respectively. LVEF was assessed by echocardiography and interpreted by cardiologists blinded to the metabolomics analysis. Data are presented as the median ± IQR. * p < 0.05 compared to the control group, # p < 0.05 compared to the HFpEF group.

Table 1 shows the baseline characteristics of the study groups. There was no significant difference in the prevalence of hypertension or diabetes between the three groups. HFpEF patients had significantly higher male to female ratio, older age, higher prevalence of dyslipidemia and atrial fibrillation than the non-HF control group. However, there was no significant difference in these characteristics between HFrEF patients and non-HF controls. Patients with HF (both HFpEF and HFrEF) had a higher body mass index, higher prevalence of CAD, and were on more cardiovascular medications than non-HF controls. There was no significant difference between the HFpEF and HFrEF groups with regard to baseline characteristics, NYHA classification, cardiovascular diseases, or the use of cardiovascular medications, with the exception that CAD was more prevalent in patients with HFrEF (Table 1). BNP and NT-proBNP levels were higher and LVEF was lower in both HF groups than in the control subjects. Similarly, the BNP and NT-proBNP levels were higher and the LVEF was lower in the HFrEF group than in the HFpEF group (Fig 2).

**Table 1:** Demographic Details of Participants.

	Control	HFpEF	HFrEF
N	38	24	20
**Baseline Characteristics**			
Male, N (%)	18 (47.37)	18 (75) *	14 (70)
Age	61.50(53.75–69.00)	67.50 *(57.50–74.75)	63.50(56.00–69.00)
**NYHA Classification**			
Class I, N(%)	NA	3 (12.5)	1 (5)
Class II, N(%)	NA	15 (62.5)	11 (55)
Class III, N(%)	NA	6 (25)	7 (35)
Class IV, N(%)	NA	0 (0)	1 (5)
**Other cardiovascular Diseases or Risk Factors**			
CAD, N(%)	5 (13.2)	10 (41.7) *	15 (75) * ^†^
Dyslipidemia, N(%)	13 (43.2)	17 (70.8) *	13 (65)
Hypertension, N(%)	22 (57.9)	15 (62.5)	10 (50)
Diabetes, N(%)	9 (23.7)	10 (41.6)	5 (25)
Atrial Fibrillation, N(%)	7 (18.4)	11 (45.8) *	5 (25)
BMI (kg/m^2^)	27.74(24.03–30.80)	31.07 * (29.13–36.13)	30.31 * (26.50–33.96)
**Cardiovascular Medications**			
Beta blockers, N(%)	8 (21.1)	21 (87.5) *	20 (100) *
ACEI or ARB, N(%)	16 (42.1)	20 (83.3) *	18 (90) *
Spironolactone, N(%)	1 (2.6)	6 (25) *	7 (35) *
Diuretic, N(%)	4 (10.5)	18 (75) *	13 (65) *
Statins, N(%)	15 (39.5)	19 (79.2) *	12 (60)
CCB, N(%)	9 (23.7)	8 (33.3)	4 (20)
Aspirin, N(%)	5 (13.2)	16 (66.7) *	15 (75) *

* p-value < 0.05 compared to control

^†^ p-value < 0.05 compared to HFpEF

HFpEF = Heart Failure with Preserved Ejection Fraction, HFrEF = Heart Failure with reduced Ejection Fraction, NYHA = New York Heart Association, CAD = Coronary Artery Disease, LVEF = Left Ventricular Ejection Fraction, BMI = Body Mass Index, BNP = B-type Natriuretic Peptide, NT-proBNP = N terminal pro-BNP, ACEI = Angiotensin Converting Enzyme Inhibitor, ARB = Angiotensin Receptor Blocker, CCB = Calcium Channel Blocker.

### Metabolomic differences between HFpEF and controls

**Fig 3:**
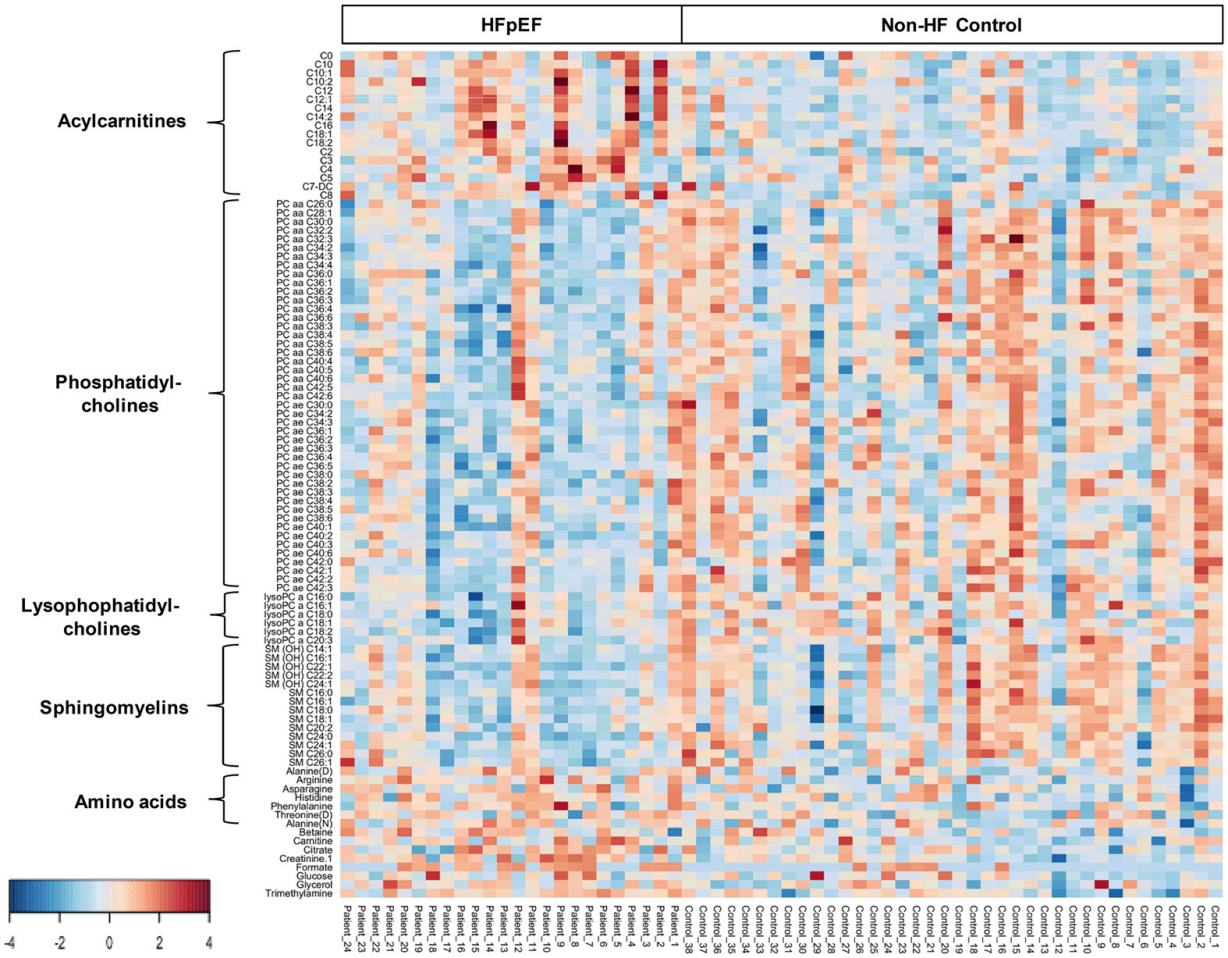
Heat map of metabolomic differences between HFpEF and controls. Heat maps were generated with the concentrations of potential candidate metabolites with univariate analysis. Similar metabolites were arranged together for use in pathway analysis through intuitive pattern discovery. The heat map displays an increase in each metabolite in relative concentration as a red color and a decrease in a metabolite as a blue color. The metabolites are listed at the left side of each row, and the subjects are shown at the bottom of each column.

Serum metabolomic analysis of a total of 181 analyzed metabolites from DI-MS (148 metabolites) and NMR (33 metabolites) revealed that the serum concentrations of short-chain, medium-chain, and long-chain acylcarnitines, carnitine, creatinine, betaine, and several amino acids were higher in HFpEF patients than non-HF controls (Fig 3, S1 Table). In addition, the serum concentrations of phosphatidylcholines (PC), lysophosphatidylcholines (LysoPC), and sphingomyelins (SM) were lower in HFpEF patients than non-HF controls (Fig 3, S1 Table).

**Fig 4:**
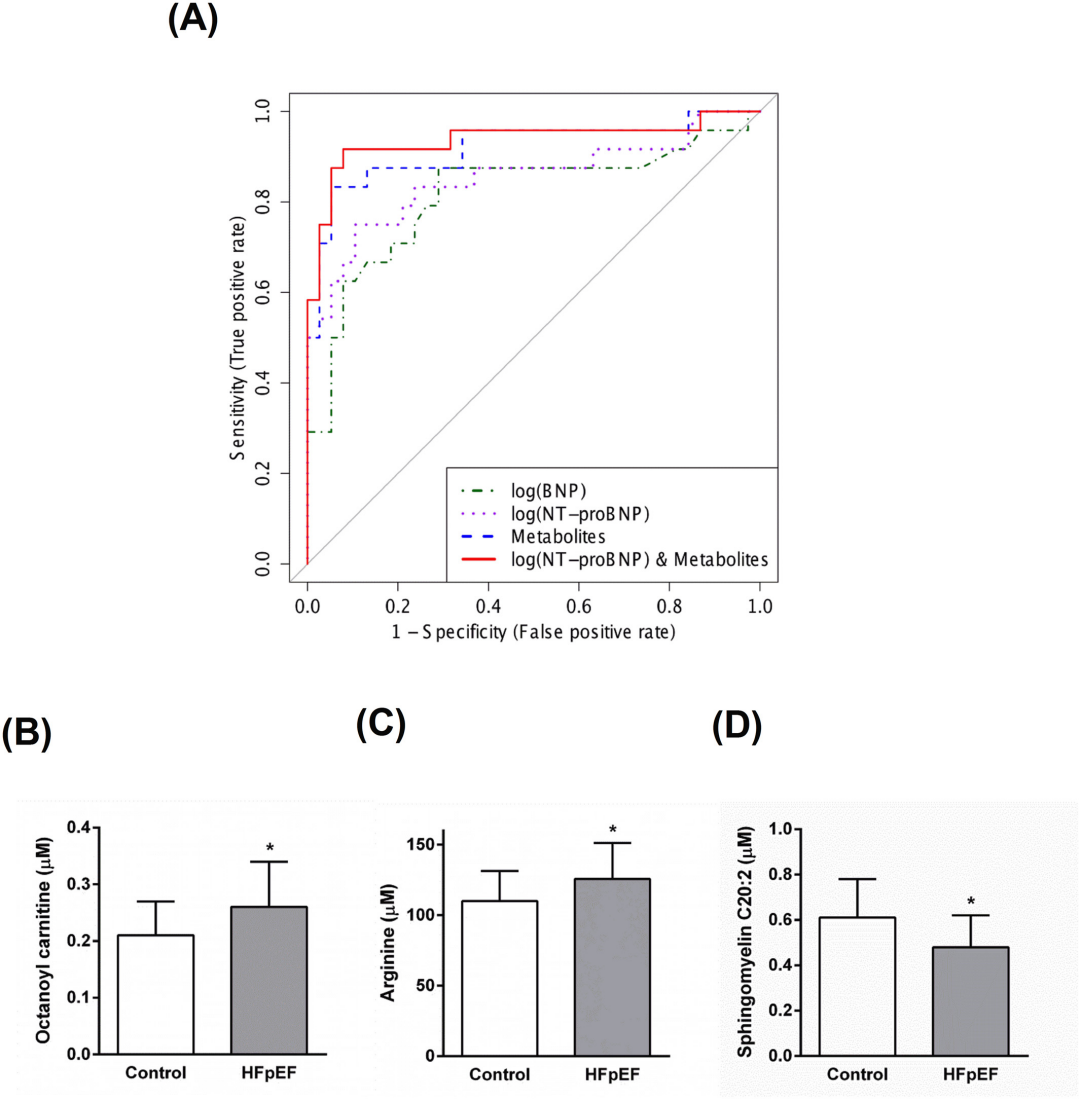
**(A) Receiver operator characteristic (ROC) analysis for serum metabolites, cardiac peptides, and combined metabolites and NT-proBNP.** Logistic regression (LR) was performed to find the most parsimonious model to discriminate HFpEF patients from control subjects using the minimum number of metabolites and/or cardiac peptides. Octanoylcarnitine, arginine, asparagine, lysophosphatidylcholine acyl C18:2, and sphingomyelin C20:2 were used in the metabolites-only panel. While octanoyl carnitine, arginine, and sphingomyelin C20:2 were used for the combined metabolites and NT-proBNP panel. **(B–D) Quantification of the metabolites used to derive the LR equation of the combined metabolites and NT-proBNP model.** Data are presented as means ± SD. * p < 0.05 compared to the control group.

To identify potential metabolite biomarkers, LR was performed to find the most parsimonious model to discriminate HFpEF patients from the non-HF controls. Since there was a significant difference in age and gender between patients with HFpEF and non-HF controls, the LR analysis was performed only for the subset of metabolites which had no significant correlation with these demographic variables. A small number of metabolites including octanoylcarnitine, arginine, asparagine, LysoPC(C18:2), and SM(C20:2) was able to discriminate HFpEF patients from the non-HF controls. Receiver operating characteristic (ROC) curve analysis using only these selected metabolites produced an area under the curve (AUC) of 0.924 (Fig 4A and Table 2). The permutation test’s result (p-value < 0.0005) for model validation indicated that the model was highly significant.

**Table 2:** Unadjusted Comparisons.

Model	AUC	Sensitivity	Specificity	NRI (95% CI)	p-value	IDI (95% CI)	p-value
**HFpEF vs. Control**							
BNP	0.816	0.750	0.763	1.276 (0.898–1.655)	< 0.0001	0.341 (0.237–0.445)	< 0.0001
NT-proBNP	0.853	0.792	0.789	0.952 (0.513–1.391)	< 0.0001	0.222 (0.122–0.321)	< 0.0001
Metabolites only	0.924	0.833	0.895	0.439 (-0.054–0.931)	0.0807	0.0807 (-0.002–0.199)	0.0547
NT-proBNP &Metabolites	0.942	0.917	0.921	-	-	-	-
**HFrEF vs. Control**							
BNP	0.942	0.850	0.842	0.355 (0.142–0.569)	0.0011	0.313 (0.193–0.433)	< 0.0001
NT-proBNP	0.991	0.950	0.947	0.153 (-0.051–0.356)	0.1421	0.103 (-0.001–0.208)	0.0527
Metabolites only	0.959	0.900	0.895	0.434 (0.208–0.661)	0.0002	0.248 (0.104–0.392)	0.0007
NT-proBNP &Metabolites	0.997	0.950	0.974	-	-	-	-
**HFpEF vs. HFrEF**							
BNP	0.727	0.650	0.667	0.95 (0.632–1.268)	< 0.0001	0.590 (0.44–0.740)	< 0.0001
NT-proBNP	0.696	0.600	0.583	0.525 (0.248–0.802)	0.0002	0.603 (0.455–0.751)	< 0.0001
Metabolites only	0.908	0.800	0.792	0.275 (0.002–0.548)	0.0484	0.248 (0.084–0.412)	0.0031
BNP & Metabolites	0.981	0.900	0.917	-	-	-	-

The NRI and IDI values are of the blended “natriuretic peptide & metabolites” model versus each individual model.

AUC = Area Under the Curve, NRI = Net Reclassification Improvement, IDI = Integrated Discrimination Improvement, CI = Confidence Interval

A separate LR model was developed incorporating a panel of three metabolites (octanoyl carnitine, arginine, and SM(C20:2) (Fig 4B–4D)) along with NT-proBNP to produce a ROC curve with an AUC of 0.942 (Fig 4A and Table 2). The permutation test’s result (p-value < 0.0005) for this model indicated that it, too, was highly significant. The biomarker models that included metabolites had higher AUCs, and higher sensitivity/specificity than models that did not include metabolites. Overall, the best model was the one that combined NT-proBNP and metabolite data (Fig 4A, Table 2, and S4 Table). However, the difference between the metabolite-only model and the metabolites+NT-proBNP model was not statistically significant an NRI of 0.439 (p = 0.0807) and an IDI of 0.098 (p = 0.0547)). On the other hand the blended (metabolites+NT-proBNP) model was significantly better than the NT-proBNP-only model (a NRI of 0.952 (p<0.0001) and IDI of 0.222 (p<0.0001)).

### Metabolomic differences between HFrEF and controls

**Fig 5:**
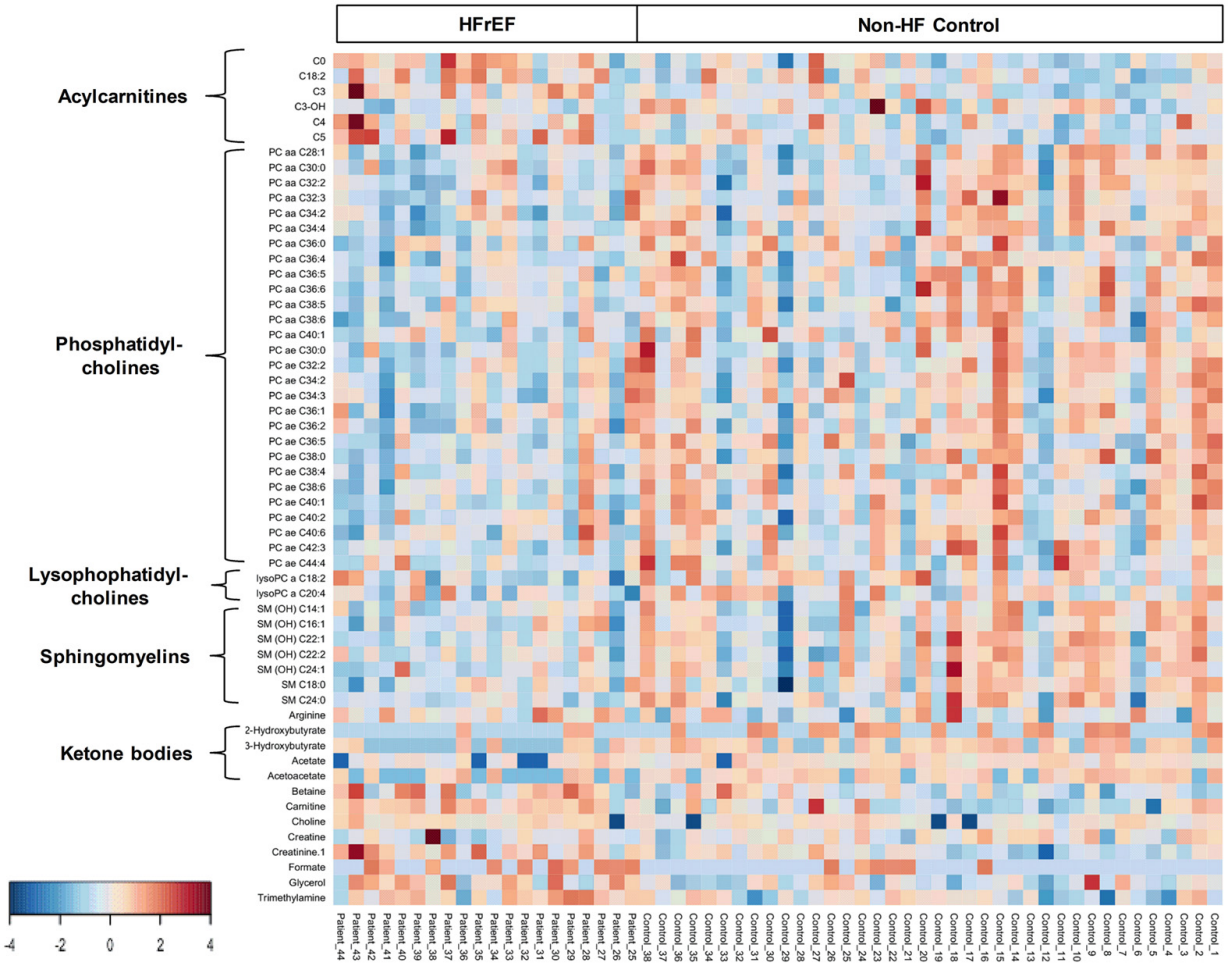
Heat map of metabolomic differences between HFrEF and controls. Heat maps were generated with the concentrations of potential candidate metabolites with univariate analysis. Similar metabolites were arranged together for use in pathway analysis through intuitive pattern discovery. The heat map displays an increase in each metabolite in relative concentration as a red color and a decrease in a metabolite as a blue color. The metabolites are listed at the left side of each row, and the subjects are shown at the bottom of each column.

The metabolomic analysis showed that the serum concentrations of some short and medium-chain acylcarnitines, carnitine, creatinine, creatine, betaine, and some amino acids were higher in HFrEF patients than non-HF controls. Whereas, the serum concentrations of several PCs, LysoPC(C18:2), LysoPC(C20:4), some sphingomyelins, acetate, acetoacetate, 2-hydroxybutyrate, and 3-hydroxybutyrate were found to be lower in HFrEF patients than non-HF controls (Fig 5, S2 Table).

**Fig 6:**
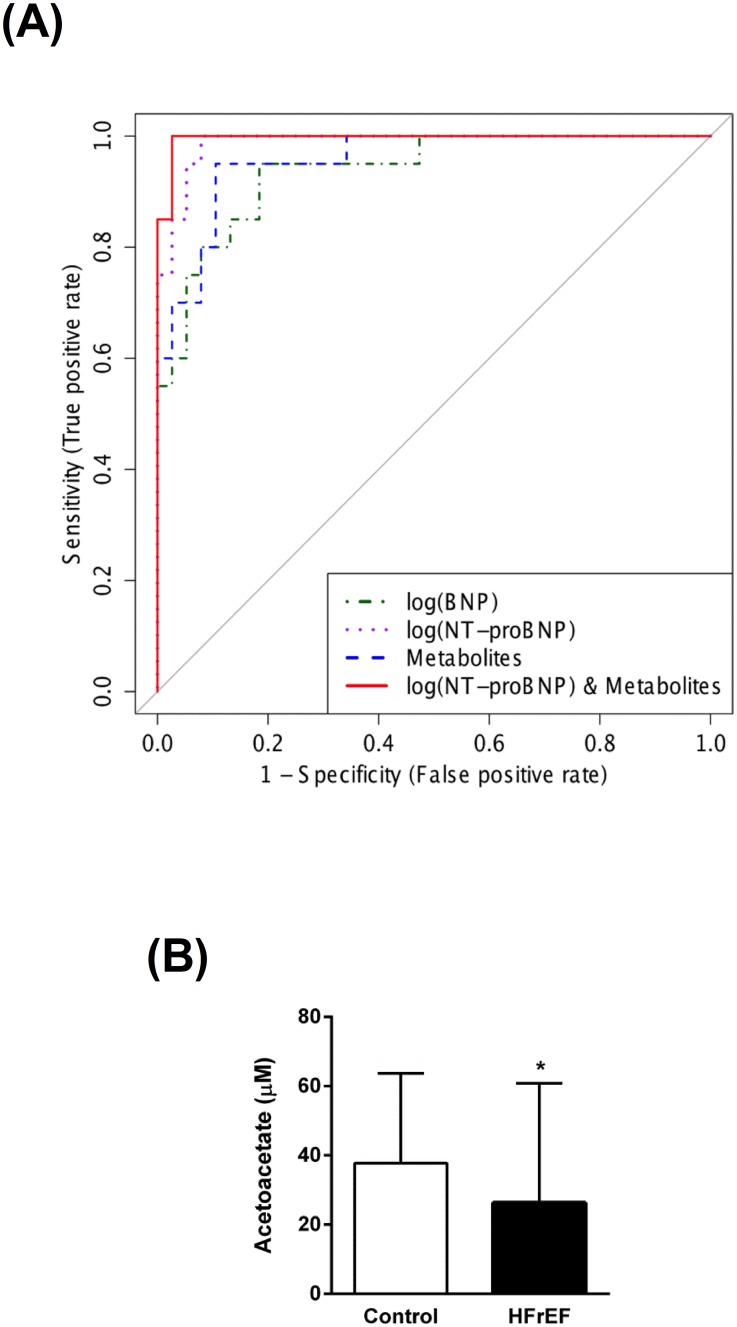
**(A) Receiver operator characteristic (ROC) analysis for serum metabolites, cardiac peptides, and combined serum metabolites and NT-proBNP.** Logistic regression (LR) was performed to find the most parsimonious model to discriminate HFrEF patients from control subjects using the minimum number of metabolites and/or cardiac peptides. Creatinine, carnitine, acetoacetate, lysophosphatidylcholine acyl C18:2, 2-hydroxybutyrate, and lysophosphatidylcholine acyl C20:4 were used in the metabolites-only panel. While acetoacetate was used for the combined metabolites and NT-proBNP panel. **(B) Quantification of acetoacetate which was used to derive the LR equation of the combined metabolites and NT-proBNP model.** Data are presented as means ± SD. * p < 0.1 compared to the control group.

For the identification of a potential biomarker panel of metabolites, we performed a similar LR analysis of metabolites from HFrEF patients and non-HF controls. Another small number of metabolites including creatinine, carnitine, acetoacetate, LysoPC(C18:2), LysoPC(C20:4), and 2-hydroxybutyrate discriminated HFrEF patients from control subjects. ROC curve analysis produced an AUC of 0.959 (Fig 6A and Table 2). An additional LR model was developed incorporating acetoacetate (Fig 6B) and NT-proBNP. ROC curve analysis of this model shows that it produced a striking AUC of 0.997 (Fig 6A). The p-value of permutation test was less than 0.0005. When all models were compared, the models that included NT-proBNP had higher AUC values than the model that used metabolites only. Nevertheless, similar to the HFpEF models, the model that combined both the NT-proBNP+metabolites achieved the highest AUC value (Fig 6A, Table 2, and S4 Table) with an NRI of 0.434 (p = 0.0002) and an IDI of 0.248 (p = 0.0007) compared to metabolites alone. However, NT-proBNP+metabolites was not significantly better than the NT-proBNP model with a NRI of 0.153 (p = 0.1421) and IDI of 0.103 (p = 0.0527).

### Metabolomic differences between HFpEF and HFrEF

**Fig 7:**
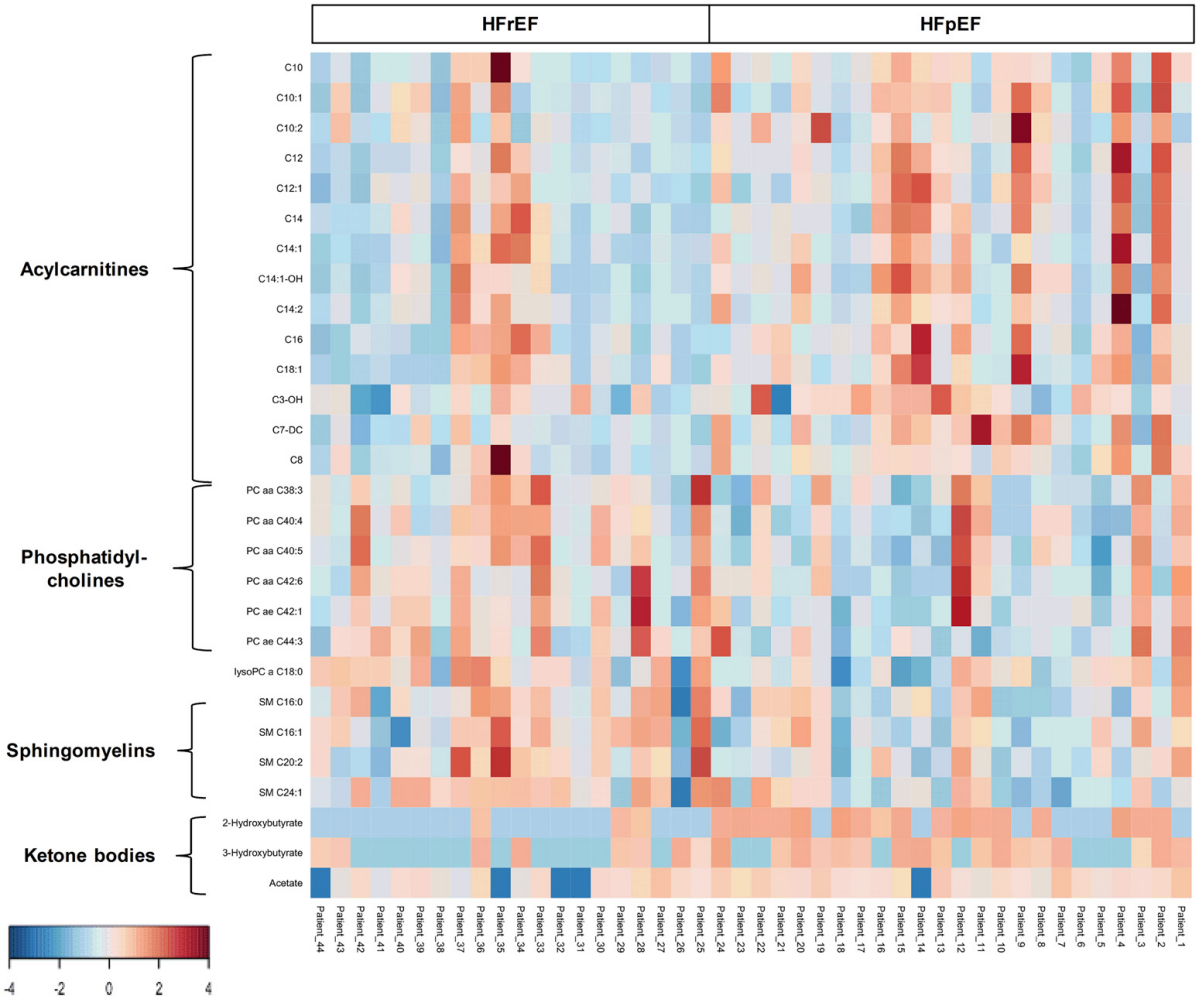
Heat map of metabolomic differences between HFpEF and HFrEF. Heat maps were generated with the concentrations of potential candidate metabolites with univariate analysis. Similar metabolites were arranged together for use in pathway analysis through intuitive pattern discovery. The heat map displays an increase in each metabolite in relative concentration as a red color and a decrease in a metabolite as a blue color. The metabolites are listed at the left side of each row, and the subjects are shown at the bottom of each column.

**Fig 8:**
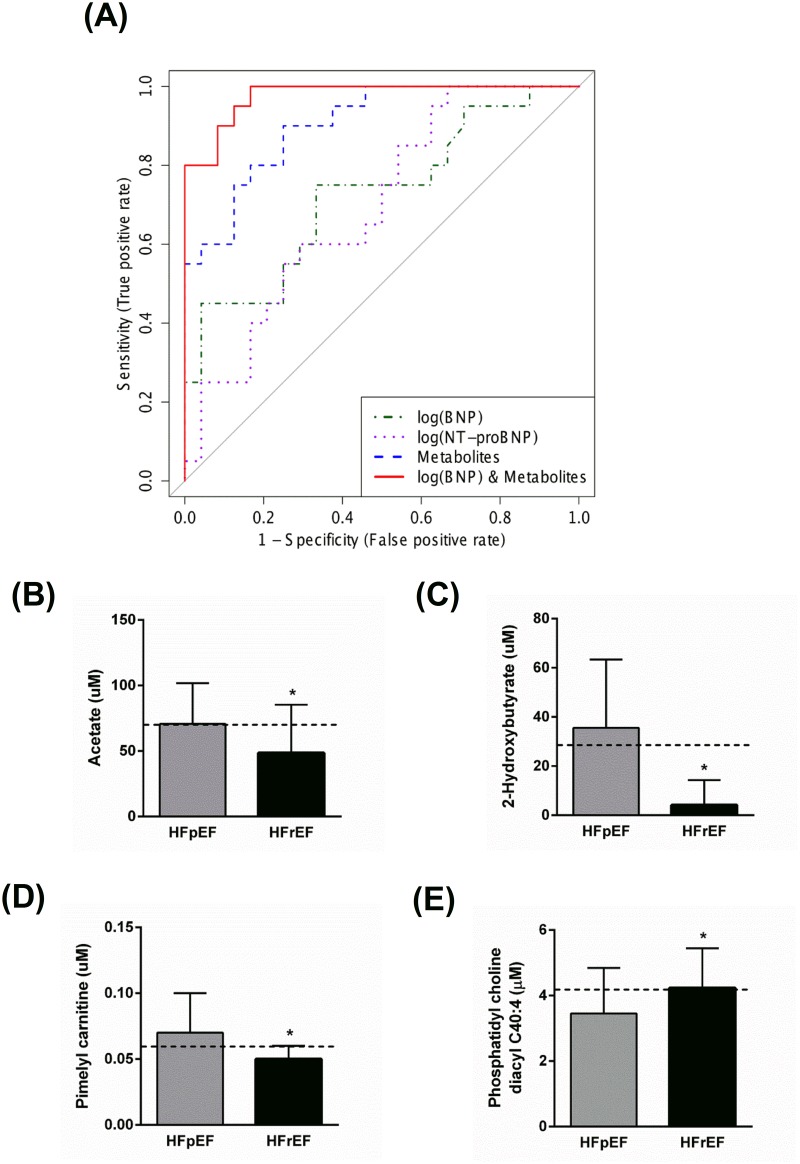
**(A) Receiver operator characteristic (ROC) analysis for serum metabolites, cardiac peptides, and combined serum metabolites and BNP.** Logistic regression (LR) was performed to find the most parsimonious model to discriminate HFpEF from HFrEF patients using the minimum number of metabolites and/or cardiac peptides. 2-hydroxybutyrate, octadecenoylcarnitine (C18:1), hydroxyprionylcarnitine (C3-OH), and sphingomyelin C24:1 were used in the metabolites-only panel. While acetate, 2-hydroxybutyrate, pimelyl carnitine, and phosphatidyl choline diacyl C40:4 were used for the combined metabolites and NT-proBNP panel. **(B–E) Quantification of the metabolites used to derive the LR equation of the combined metabolites and NT-proBNP model.** Data are presented as means ± SD. The dashed line represents the metabolite concentration in control subjects. * p < 0.05 compared to the HFpEF group.

For the metabolomic data, long-chain acylcarnitines, 2-hydroxybutyrate, 3-hydroxybutyrate, and acetate were found to be higher in the HFpEF group than the HFrEF group, while SM(C24:1), some PCs and LysoPCs were found to be lower in the HFpEF group than the HFrEF group (Fig 7 and S3 Table). In order to identify a panel of selected metabolites that can discriminate between HFpEF and HFrEF, we performed a similar LR analysis. A panel of 4 metabolites (2-hydroxybutyrate, octadecenoylcarnitine (C18:1), hydroxyprionylcarnitine (C3-OH), and SM(C24:1)) was identified that discriminated between HFpEF and HFrEF. Using only these metabolites, ROC curve analysis produced an AUC of 0.908 (Fig 8A and Table 2). Permutation testing (p-value < 0.0185) for model validation indicated that the model was statistically significant.

LR including the metabolites and natriuretic peptides was also performed. This model utilized acetate (Fig 8B), 2-hydroxybutyrate (Fig 8C), pimelylcarnitine (Fig 8D), and PC(C40:4) (Fig 8D) as the metabolites and BNP. ROC curve analysis of this model produced an AUC of 0.981 (Fig 8A) with a permutation test p-value of <0.0005. As before, the model that combined BNP with the metabolites achieved the highest AUC value (Fig 8A, Table 2, and S4 Table). This blended model had a NRI of 0.275 (p = 0.0484) and an IDI of 0.248 (p = 0.0031) compared to metabolites alone and a NRI of 0.95 (p<0.00001) and an IDI of 0.569 (p<0.00001) compared to BNP alone.

## Discussion

Several potential metabolic perturbations have been discovered in HF patients in the current study. Although some of these metabolite profile differences were observed in both HFpEF and HFrEF, there are several pathways that are differentially altered in HFpEF versus HFrEF. For instance, we discovered that serum concentrations of short-chain acylcarnitines are higher in both HFpEF and HFrEF patients than non-HF controls, while medium and long-chain acylcarnitines were higher in HFpEF patients than both HFrEF patients and non-HF controls. Carnitine and its acyl derivatives play a key role in fatty acid uptake and mitochondrial metabolism [23], hence an increase in acylcarnitines may imply inefficient β-oxidation [24] in HFpEF patients. In addition, several reports have demonstrated the detrimental effects of long-chain acylcarnitines, as they are pro-inflammatory [25] and arrhythmogenic [26]. A recent report has shown that plasma levels of long-chain acylcarnitines predict cardiovascular mortality in dialysis patients [27]. Therefore, high serum concentration of long-chain acylcarnitines in HFpEF patients may corroborate the findings that show inflammation is strongly associated with diastolic dysfunction [27]. It may also explain the recent findings that the expression of the pro-inflammatory tumor necrosis factor-alpha receptor 2 (TNFR2) is higher in HFpEF than in HFrEF patients [28]. Moreover, due to their arrhythmogenic properties, higher concentrations of long-chain acylcarnitines may explain the high prevalence of atrial fibrillation among HFpEF patients, as observed in our cohort and in other studies [29].

In addition to increases in acyl derivatives in HFpEF compared to HFrEF, the serum carnitine levels were also higher in both HFpEF and HFrEF patients compared to controls. Since the myocardial content of carnitine tends to be lower in HF patients [30], higher serum carnitine level can be attributed to a leakage of carnitine from the myocardium to the blood. Our findings are in agreement with other studies showing that plasma carnitine levels are higher in HF patients than controls [31].

Several other metabolic perturbations were observed in our patient cohorts that warrant discussion. For instance, the serum concentrations of creatinine and several amino acids are higher in both HFpEF and HFrEF patients than non-HF controls. The higher creatinine concentration in HF patients may be secondary to the renal impairment commonly observed in HF patients [32], while the increase in the amino acids may imply a hypercatabolic state that has been reported in chronic HF patients [12, 33]. Conversely, serum concentrations of PCs, LysoPCs, and SM were lower in HFpEF and HFrEF patients compared to controls. The decrease in these phospholipids along with an increase in the serum concentration of betaine may indicate a shift in choline metabolism toward more betaine and less PC production. This is of potential interest as choline deficiency has been reported to cause cardiac dysfunction [34] and may be involved in the pathogenesis of HF. These findings are consistent with animal models, where PCs and other phospholipids were found to be lower in heart tissues from Syrian hamsters with hereditary cardiomyopathy compared to normal hamsters [35]. Together, these findings highlight the potential role of choline metabolism in the pathogenesis of both HFpEF and HFrEF.

Other metabolic abnormalities were observed in HFrEF patients only. For instance, creatine levels were found to be higher in HFrEF patients than controls. Phosphocreatine is converted to creatine to produce ATP which is an essential energy source for myocardial contraction [36]; therefore, increased serum creatine levels may indicate a state of energy depletion. Notably, two previous metabolomics studies have demonstrated higher serum creatine levels in HF patients [12, 37].

In contrast to creatine, the serum concentrations of the ketone bodies, acetoacetate, 2-hydroxybutyrate and 3-hydroxybutyrate were lower in HFrEF patients than both HFpEF patients and non-HF controls. This may imply a greater reliance on ketone bodies as an energy source in HFrEF patients. In contrast to our results, older studies have shown that blood ketone bodies are higher in HF patients than in healthy control subjects after 8–12 hr fasting [38, 39]. More recently, it has been shown that there was no significant difference in plasma 2-hydroxybutyrate levels between HF patients without diabetes and controls [40]. Fasting, the severity of HF, and other co-morbidities may be the reason for the varying results. However, metabolomic studies cannot determine whether a metabolite is reduced because of decreased production, increased degradation and/or uptake, or both [41]. Neither can we be certain on what organ(s) are responsible for the changes observed in the serum of these patients. Further research is warranted to elucidate the mechanisms of the metabolic perturbations reported in this study.

Since we have identified distinct metabolomic fingerprints of HFpEF and HFrEF, we sought to use these fingerprints to discover novel panels of biomarkers that can be used to help differentiate between HFpEF and HFrEF. Although the cardiac peptides, BNP and NT-proBNP, are considered gold standards for the diagnosis of HF [42, 43], the sensitivity and specificity of these cardiac peptides can be significantly affected by several confounding factors including age, obesity, sex, as well as pulmonary, hepatic, and renal dysfunction [14]. Consequently, the utility of cardiac peptides as biomarkers to identify HFpEF patients is questionable. While some studies have shown that these peptides can distinguish HFpEF patients from controls [14, 44–47], other studies have demonstrated that these peptides are not reliable enough for the diagnosis of HFpEF [48–50]. However, using only three metabolites (octanoyl carnitine, arginine, and SM(C20:2)) along with NT-proBNP, we discovered a novel panel of biomarkers that reliably distinguishes HFpEF from control subjects. Using this panel, we obtained a ROC AUC of 0.942. Of interest, a panel of 3 myocardial matrix biomarkers (MMP-2, TIMP-4, and MMP-8) has been combined with clinical variables to produce a ROC AUC of 0.79 for diagnosing HFpEF [14]. Similarly, combining BNP and MMP-2 has been shown to diagnose HFpEF with 0.81 sensitivity and 0.83 specificity [44]. In addition, soluble ST2 (suppression of tumorigenicity 2, a blood protein confirmed to act as a decoy receptor for interleukin-33) has been shown to be a potential biomarker for HFpEF with an AUC of 0.80 for its ROC curve [45]. In comparison, our newly identified panel of metabolites far exceeds the accuracy of existing panels of protein biomarkers and may have potential to be used clinically in the diagnosis of HFpEF.

In contrast to the diagnosis of HFpEF, the standard cardiac peptides produced very high ROC AUCs in our study, suggesting that these cardiac peptides are reliable biomarkers to distinguish HFrEF patients from controls. Nevertheless, LR analysis of the metabolites combined with the standard cardiac peptides revealed that measuring only one metabolite (acetoacetate) along with the NT-proBNP increased the AUC of the ROC curve from 0.991 for NT-proBNP alone to 0.997 for the acetoacetate plus the NT-proBNP. Taking into account the confounding factors that may limit the diagnostic utility of these cardiac peptides [14], the addition of measuring acetoacetate along with plasma natriuretic peptides may be of clinical importance; although they still cannot be used to accurately differentiate between HFrEF and HFpEF patients [51]. In fact, BNP and NT-proBNP produced ROC curves with relatively low AUC values of 0.727 and 0.696 for BNP and NT-proBNP, respectively, when used to distinguish between HFpEF and HFrEF patients in our cohort. However, we identified a novel panel of 4 metabolites that when used along with BNP, produced a ROC AUC value of 0.981 to distinguish HFrEF patients from HFpEF patients. To the best of our knowledge, this is the first report to identify a set of biomarkers that can differentiate between HFpEF and HFrEF patients with such a high level of accuracy.

Several limitations to our current study warrant further discussion. First, a relatively small number of patients were enrolled in each HF group. Therefore, the metabolites identified in this study need to be verified by larger, prospective, validation cohort. Nevertheless, the high ROC AUC values for this small patient number suggest the high potential of these metabolites to play an important role in HF diagnosis. In addition, the metabolomic profile is a collective snapshot of all the metabolic perturbations that may include confounding factors such as other disease states, acute illnesses, or medication use [12]. Therefore, we cannot be certain that the observed metabolic perturbations are solely attributable to the HFpEF or the HFrEF syndrome. Of interest, there was no significant difference in all the demographic and clinical data between the HFpEF and the HFrEF groups except that CAD was more prevalent in HFrEF group than in the HFpEF group. Although this may represent a confounding factor, it is consistent with the overall epidemiology of HFpEF and HFrEF [52]. However, the metabolites that differentiate between HFpEF and HFrEF groups in the current study were adjusted to account for the difference in the prevalence of CAD between the two groups. As such, the metabolic differences between HFpEF and HFrEF patients cannot be attributed to any demographic, medication, or other disease state factors.

In conclusion, this study is the first to investigate the metabolomic profile of HFpEF and HFrEF patients compared to non-HF controls. The unbiased and systematic approach employed in this metabolomic study enabled us to identify a metabolic fingerprint of both HFpEF and HFrEF in addition to three panels of metabolites that can be used to diagnose these conditions. We chose a LR model to ensure that our results would have clear clinical utility. This is because LR produces a simple equation with a defined threshold that can be used to classify individuals. Therefore, once our findings are externally validated and refined, our work may allow a simple decision tree that would aid in the rapid diagnosis of HFpEF. In summary, we have used quantitative metabolomics to identify a novel panel of blood-based metabolites that when used in combination with natriuretic peptides, can accurately identify HFpEF patients and distinguish between HFpEF and HFrEF patients.

## Supporting Information

S1 Table Univariate Analysis of HFpEF vs. Control. (DOCX)

S2 Table Univariate Analysis of HFrEF vs. Control. (DOCX)

S3 Table Univariate analysis of HFrEF vs. HFpEF. (DOCX)

S4 Table Logistic regression Analyses for the Models. (DOCX)
