# Supplementary material for: Metabolomic Fingerprint of Heart Failure with Preserved Ejection Fraction
Source: PLoS One. 2015 May 26;10(5):e0124844. doi: 10.1371/journal.pone.0124844 (PMC4444296; doi:10.1371/journal.pone.0124844)
Supplement: S1 Table — (DOCX) [file pone.0124844.s001.docx]

**S1Table. Univariate Analysis of HFpEF vs. Control.**

| **Metabolite** | **p-value** | **Mean(SD)** | | **HFpEF / Control** | **Fold Change** |
| --- | --- | --- | --- | --- | --- |
|  |  | **HFpEF** | **Control** |  |  |
| Number of cases | - | 24 | 38 | - | - |
| BNP | 0.0000 | 154.62(159.64) | 36.61(48.27) | Up | 4.22 |
| NT.pro.BNP | 0.0000 | 110.05(139.64) | 9.45(11.5) | Up | 11.65 |
| C0 | 0.0855 | 50.65(14.14) | 43.57(9.97) | Up | 1.16 |
| C10 | 0.0149 | 0.31(0.13) | 0.23(0.09) | Up | 1.32 |
| C10.1 | 0.0050 | 0.28(0.09) | 0.22(0.06) | Up | 1.29 |
| C10.2 | 0.0091 | 0.07(0.03) | 0.05(0.02) | Up | 1.37 |
| C12 | 0.0021 | 0.15(0.07) | 0.1(0.04) | Up | 1.5 |
| C12.1 | 0.0171 | 0.29(0.1) | 0.23(0.07) | Up | 1.25 |
| C14 | 0.0312 | 0.05(0.01) | 0.05(0.01) | Up | 1.18 |
| C14.2 | 0.0131 | 0.04(0.03) | 0.03(0.01) | Up | 1.52 |
| C16 | 0.0215 | 0.14(0.04) | 0.12(0.03) | Up | 1.19 |
| C18.1 | 0.0014 | 0.2(0.08) | 0.14(0.04) | Up | 1.39 |
| C18.2 | 0.0039 | 0.07(0.03) | 0.05(0.02) | Up | 1.35 |
| C2 | 0.0103 | 10.28(3.49) | 8.05(3.22) | Up | 1.28 |
| C3 | 0.0084 | 0.58(0.2) | 0.44(0.14) | Up | 1.31 |
| C4 | 0.0867 | 0.33(0.18) | 0.26(0.1) | Up | 1.3 |
| C5 | 0.0200 | 0.2(0.06) | 0.16(0.04) | Up | 1.22 |
| C7.DC | 0.0237 | 0.07(0.03) | 0.06(0.02) | Up | 1.26 |
| C8 | 0.0041 | 0.26(0.08) | 0.21(0.06) | Up | 1.29 |
| PC.aa.C26.0 | 0.0908 | 2.84(0.5) | 3.08(0.48) | Down | -1.08 |
| PC.aa.C28.1 | 0.0161 | 3.25(0.79) | 3.87(1.01) | Down | -1.19 |
| PC.aa.C30.0 | 0.0324 | 4.01(1.5) | 5.12(2.19) | Down | -1.28 |
| PC.aa.C32.2 | 0.0097 | 4.08(1.91) | 5.75(3.77) | Down | -1.41 |
| PC.aa.C32.3 | 0.0027 | 0.61(0.18) | 0.83(0.35) | Down | -1.37 |
| PC.aa.C34.2 | 0.0013 | 342.12(78.53) | 409.32(87.37) | Down | -1.2 |
| PC.aa.C34.3 | 0.0073 | 20.04(6.27) | 25.87(9.06) | Down | -1.29 |
| PC.aa.C34.4 | 0.0117 | 2.03(0.81) | 2.76(1.13) | Down | -1.36 |
| PC.aa.C36.0 | 0.0842 | 2.58(0.89) | 3.14(1.2) | Down | -1.22 |
| PC.aa.C36.1 | 0.0161 | 49.24(14.45) | 60.62(19.39) | Down | -1.23 |
| PC.aa.C36.2 | 0.0040 | 220.75(59.14) | 267.03(66.1) | Down | -1.21 |
| PC.aa.C36.3 | 0.0137 | 138.91(39.01) | 165.5(44.39) | Down | -1.19 |
| PC.aa.C36.4 | 0.0046 | 189.52(48.48) | 228.74(50.69) | Down | -1.21 |
| PC.aa.C36.6 | 0.0265 | 1.26(0.59) | 1.7(0.84) | Down | -1.36 |
| PC.aa.C38.3 | 0.0937 | 54.02(19.15) | 62.34(20.77) | Down | -1.15 |
| PC.aa.C38.4 | 0.0318 | 126.98(34.03) | 147.79(39.15) | Down | -1.16 |
| PC.aa.C38.5 | 0.0085 | 68.83(20.51) | 81.9(19.68) | Down | -1.19 |
| PC.aa.C38.6 | 0.0285 | 85.74(28.21) | 103.41(31.68) | Down | -1.21 |
| PC.aa.C40.4 | 0.0171 | 3.45(1.39) | 4.16(1.34) | Down | -1.21 |
| PC.aa.C40.5 | 0.0181 | 11.44(4.62) | 13.85(4.15) | Down | -1.21 |
| PC.aa.C40.6 | 0.0731 | 31.04(10.53) | 36.08(11.41) | Down | -1.16 |
| PC.aa.C42.5 | 0.0233 | 0.38(0.12) | 0.45(0.13) | Down | -1.18 |
| PC.aa.C42.6 | 0.0233 | 0.71(0.17) | 0.79(0.16) | Down | -1.12 |
| PC.ae.C30.0 | 0.0664 | 0.46(0.14) | 0.53(0.18) | Down | -1.16 |
| PC.ae.C34.2 | 0.0155 | 10.77(3.28) | 13.46(4.65) | Down | -1.25 |
| PC.ae.C34.3 | 0.0043 | 6.96(2.42) | 9.36(3.55) | Down | -1.35 |
| PC.ae.C36.1 | 0.0275 | 8.74(2.68) | 10.03(2.51) | Down | -1.15 |
| PC.ae.C36.2 | 0.0188 | 15.39(4.72) | 18.32(4.96) | Down | -1.19 |
| PC.ae.C36.3 | 0.0155 | 8.14(2.21) | 9.97(3.03) | Down | -1.22 |
| PC.ae.C36.4 | 0.0260 | 18.2(5.92) | 22.08(7.25) | Down | -1.21 |
| PC.ae.C36.5 | 0.0155 | 12.7(4.09) | 16.05(5.43) | Down | -1.26 |
| PC.ae.C38.0 | 0.0220 | 2.55(0.97) | 3.16(1.03) | Down | -1.24 |
| PC.ae.C38.2 | 0.0095 | 1.89(0.64) | 2.19(0.54) | Down | -1.16 |
| PC.ae.C38.3 | 0.0546 | 4.12(1.26) | 4.76(1.31) | Down | -1.16 |
| PC.ae.C38.4 | 0.0196 | 14.16(4.09) | 16.25(4.37) | Down | -1.15 |
| PC.ae.C38.5 | 0.0841 | 19.76(4.5) | 22.6(6.07) | Down | -1.14 |
| PC.ae.C38.6 | 0.0192 | 7.98(2.19) | 9.88(2.98) | Down | -1.24 |
| PC.ae.C40.1 | 0.0009 | 1.27(0.37) | 1.66(0.43) | Down | -1.31 |
| PC.ae.C40.2 | 0.0124 | 1.64(0.46) | 1.91(0.44) | Down | -1.16 |
| PC.ae.C40.3 | 0.0643 | 0.95(0.24) | 1.09(0.29) | Down | -1.14 |
| PC.ae.C40.6 | 0.0965 | 5.48(1.18) | 6.31(1.81) | Down | -1.15 |
| PC.ae.C42.0 | 0.0603 | 0.62(0.08) | 0.67(0.11) | Down | -1.08 |
| PC.ae.C42.1 | 0.0020 | 0.32(0.09) | 0.41(0.12) | Down | -1.25 |
| PC.ae.C42.2 | 0.0381 | 0.56(0.13) | 0.64(0.16) | Down | -1.13 |
| PC.ae.C42.3 | 0.0895 | 0.79(0.2) | 0.92(0.27) | Down | -1.15 |
| lysoPC.a.C16.0 | 0.0091 | 73.15(13.93) | 83.48(14.67) | Down | -1.14 |
| lysoPC.a.C16.1 | 0.0519 | 2.65(1.12) | 2.88(0.88) | Down | -1.09 |
| lysoPC.a.C18.0 | 0.0039 | 21.6(5.06) | 26.22(5.93) | Down | -1.21 |
| lysoPC.a.C18.1 | 0.0510 | 21.15(5.32) | 23.83(5.62) | Down | -1.13 |
| lysoPC.a.C18.2 | 0.0011 | 28.23(8.39) | 37.27(10.8) | Down | -1.32 |
| lysoPC.a.C20.3 | 0.0025 | 2.2(0.79) | 2.64(0.72) | Down | -1.2 |
| SM..OH..C14.1 | 0.0140 | 6.17(1.44) | 7.29(2.03) | Down | -1.18 |
| SM..OH..C16.1 | 0.0089 | 3.25(0.85) | 3.81(0.93) | Down | -1.17 |
| SM..OH..C22.1 | 0.0033 | 10.43(3.64) | 13.58(4.02) | Down | -1.3 |
| SM..OH..C22.2 | 0.0087 | 9.22(2.14) | 11.18(3.13) | Down | -1.21 |
| SM..OH..C24.1 | 0.0164 | 1.1(0.37) | 1.34(0.42) | Down | -1.22 |
| SM.C16.0 | 0.0114 | 95.36(14.27) | 109.56(23.62) | Down | -1.15 |
| SM.C16.1 | 0.0168 | 15(3) | 17.62(4.33) | Down | -1.17 |
| SM.C18.0 | 0.0039 | 20.62(4.81) | 24.84(6.29) | Down | -1.2 |
| SM.C18.1 | 0.0027 | 10.18(2.2) | 12.31(3.1) | Down | -1.21 |
| SM.C20.2 | 0.0045 | 0.48(0.14) | 0.61(0.17) | Down | -1.26 |
| SM.C24.0 | 0.0083 | 18.93(6.71) | 23.76(6.55) | Down | -1.25 |
| SM.C24.1 | 0.0053 | 45.07(8.14) | 53.21(13.01) | Down | -1.18 |
| SM.C26.0 | 0.0453 | 0.14(0.05) | 0.17(0.06) | Down | -1.25 |
| SM.C26.1 | 0.0469 | 0.32(0.12) | 0.37(0.1) | Down | -1.15 |
| Alanine.D. | 0.0438 | 500.88(107.01) | 444.61(116.67) | Up | 1.13 |
| Arginine | 0.0083 | 125.77(25.5) | 110.11(21.24) | Up | 1.14 |
| Asparagine | 0.0394 | 69.29(13.01) | 61.7(12.09) | Up | 1.12 |
| Histidine | 0.0164 | 80.88(11.04) | 73.51(11.42) | Up | 1.1 |
| Phenylalanine | 0.0574 | 76.83(15.96) | 70(10.98) | Up | 1.1 |
| Threonine.D. | 0.0401 | 133.92(29.45) | 117.95(31.75) | Up | 1.14 |
| Alanine.N. | 0.0936 | 496.62(101.29) | 448.89(115.71) | Up | 1.11 |
| Betaine | 0.0423 | 62.92(17.18) | 54.77(15.5) | Up | 1.15 |
| Carnitine | 0.0242 | 47.22(15.19) | 39.33(13.25) | Up | 1.2 |
| Citrate | 0.0480 | 144.54(44.65) | 122.3(27.85) | Up | 1.18 |
| Creatinine.1 | 0.0012 | 140.68(40.76) | 108.59(21.94) | Up | 1.3 |
| Formate | 0.0021 | 33.48(33.2) | 11.84(29.81) | Up | 2.83 |
| Glucose | 0.0514 | 5535.11(1872.49) | 4894.19(1723.06) | Up | 1.13 |
| Glycerol | 0.0432 | 346.48(169.89) | 284.6(213.9) | Up | 1.22 |
| Trimethylamine | 0.0030 | 32.36(6.19) | 26.55(7.07) | Up | 1.22 |
