# Supplementary material for: Metabolomic Fingerprint of Heart Failure with Preserved Ejection Fraction
Source: PLoS One. 2015 May 26;10(5):e0124844. doi: 10.1371/journal.pone.0124844 (PMC4444296; doi:10.1371/journal.pone.0124844)
Supplement: S2 Table — (DOCX) [file pone.0124844.s002.docx]

**S2 Table. Univariate Analysis of HFrEF vs. Control.**

| **Metabolite** | **p-value** | **Mean(SD)** | | **HFrEF /  Control** | **Fold Change** |
| --- | --- | --- | --- | --- | --- |
|  |  | **HFrEF** | **Control** |  |  |
| Number of cases | - | 20 | 38 | - |  |
| BNP | 0.0000 | 367.5(306.25) | 36.61(48.27) | Up | 10.04 |
| NT.pro.BNP | 0.0000 | 238.43(294.38) | 9.45(11.5) | Up | 25.24 |
| C0 | 0.0011 | 54.38(13.17) | 43.57(9.97) | Up | 1.25 |
| C18.2 | 0.0798 | 0.06(0.02) | 0.05(0.02) | Up | 1.19 |
| C3 | 0.0039 | 0.63(0.32) | 0.44(0.14) | Up | 1.44 |
| C3.OH | 0.0200 | 0.06(0.01) | 0.07(0.01) | Down | -1.11 |
| C4 | 0.0425 | 0.33(0.14) | 0.26(0.1) | Up | 1.28 |
| C5 | 0.0107 | 0.22(0.08) | 0.16(0.04) | Up | 1.35 |
| PC.aa.C28.1 | 0.0240 | 3.23(0.6) | 3.87(1.01) | Down | -1.2 |
| PC.aa.C30.0 | 0.0732 | 4.18(1.8) | 5.12(2.19) | Down | -1.23 |
| PC.aa.C32.2 | 0.0141 | 3.88(2.09) | 5.75(3.77) | Down | -1.48 |
| PC.aa.C32.3 | 0.0321 | 0.67(0.29) | 0.83(0.35) | Down | -1.24 |
| PC.aa.C34.2 | 0.0120 | 348.4(107.39) | 409.32(87.37) | Down | -1.17 |
| PC.aa.C34.4 | 0.0015 | 1.87(0.56) | 2.76(1.13) | Down | -1.47 |
| PC.aa.C36.0 | 0.0669 | 2.57(0.85) | 3.14(1.2) | Down | -1.22 |
| PC.aa.C36.4 | 0.0014 | 185.9(31.92) | 228.74(50.69) | Down | -1.23 |
| PC.aa.C36.5 | 0.0669 | 34.81(10.82) | 46.61(21.41) | Down | -1.34 |
| PC.aa.C36.6 | 0.0022 | 1.1(0.3) | 1.7(0.84) | Down | -1.56 |
| PC.aa.C38.5 | 0.0525 | 72.19(13.9) | 81.9(19.68) | Down | -1.13 |
| PC.aa.C38.6 | 0.0073 | 79.2(25.38) | 103.41(31.68) | Down | -1.31 |
| PC.aa.C40.1 | 0.0759 | 0.39(0.09) | 0.44(0.12) | Down | -1.13 |
| PC.ae.C30.0 | 0.0272 | 0.43(0.14) | 0.53(0.18) | Down | -1.23 |
| PC.ae.C32.2 | 0.0525 | 0.8(0.24) | 0.93(0.29) | Down | -1.17 |
| PC.ae.C34.2 | 0.0450 | 10.96(3.64) | 13.46(4.65) | Down | -1.23 |
| PC.ae.C34.3 | 0.0220 | 7.51(3.37) | 9.36(3.55) | Down | -1.25 |
| PC.ae.C36.1 | 0.0745 | 8.79(2.44) | 10.03(2.51) | Down | -1.14 |
| PC.ae.C36.2 | 0.0745 | 15.59(5.14) | 18.32(4.96) | Down | -1.17 |
| PC.ae.C36.5 | 0.0296 | 13(3.55) | 16.05(5.43) | Down | -1.23 |
| PC.ae.C38.0 | 0.0031 | 2.39(0.6) | 3.16(1.03) | Down | -1.32 |
| PC.ae.C38.4 | 0.0903 | 14.37(3.18) | 16.25(4.37) | Down | -1.13 |
| PC.ae.C38.6 | 0.0197 | 8.08(2.19) | 9.88(2.98) | Down | -1.22 |
| PC.ae.C40.1 | 0.0017 | 1.33(0.33) | 1.66(0.43) | Down | -1.25 |
| PC.ae.C40.2 | 0.0800 | 1.71(0.41) | 1.91(0.44) | Down | -1.12 |
| PC.ae.C40.6 | 0.0985 | 5.57(1.86) | 6.31(1.81) | Down | -1.13 |
| PC.ae.C42.3 | 0.0694 | 0.79(0.16) | 0.92(0.27) | Down | -1.16 |
| PC.ae.C44.4 | 0.0844 | 0.4(0.1) | 0.46(0.15) | Down | -1.16 |
| lysoPC.a.C18.2 | 0.0496 | 31.52(11.38) | 37.27(10.8) | Down | -1.18 |
| lysoPC.a.C20.4 | 0.0889 | 7.06(2.24) | 8.21(2.2) | Down | -1.16 |
| SM..OH..C14.1 | 0.0773 | 6.32(1.4) | 7.29(2.03) | Down | -1.15 |
| SM..OH..C16.1 | 0.0829 | 3.39(0.86) | 3.81(0.93) | Down | -1.13 |
| SM..OH..C22.1 | 0.0012 | 10.44(1.64) | 13.58(4.02) | Down | -1.3 |
| SM..OH..C22.2 | 0.0669 | 9.76(2.06) | 11.18(3.13) | Down | -1.15 |
| SM..OH..C24.1 | 0.0064 | 1.09(0.31) | 1.34(0.42) | Down | -1.23 |
| SM.C18.0 | 0.0873 | 22.2(5.76) | 24.84(6.29) | Down | -1.12 |
| SM.C24.0 | 0.0019 | 19.38(2.39) | 23.76(6.55) | Down | -1.23 |
| Arginine | 0.0933 | 118.19(17.47) | 110.11(21.24) | Up | 1.07 |
| X2.Hydroxybutyrate | 0.0078 | 4.14(10.18) | 26.9(32.92) | Down | -6.49 |
| X3.Hydroxybutyrate | 0.0147 | 29.61(44.27) | 64.88(60.4) | Down | -2.19 |
| Acetate | 0.0301 | 48.55(36.76) | 69.14(30.49) | Down | -1.42 |
| Acetoacetate | 0.0805 | 26.27(34.57) | 37.75(25.96) | Down | -1.44 |
| Betaine | 0.0506 | 70.68(28.54) | 54.77(15.5) | Up | 1.29 |
| Carnitine | 0.0001 | 50.38(8.11) | 39.33(13.25) | Up | 1.28 |
| Choline | 0.0162 | 20.49(8.21) | 15.99(6.72) | Up | 1.28 |
| Creatine | 0.0468 | 121.89(372.94) | 50.77(21.53) | Up | 2.4 |
| Creatinine.1 | 0.0002 | 148.57(48.24) | 108.59(21.94) | Up | 1.37 |
| Formate | 0.0103 | 34.2(42.65) | 11.84(29.81) | Up | 2.89 |
| Glycerol | 0.0054 | 436.26(235.59) | 284.6(213.9) | Up | 1.53 |
| Trimethylamine | 0.0013 | 34.06(8.59) | 26.55(7.07) | Up | 1.28 |
