# Supplementary material for: Metabolomic Fingerprint of Heart Failure with Preserved Ejection Fraction
Source: PLoS One. 2015 May 26;10(5):e0124844. doi: 10.1371/journal.pone.0124844 (PMC4444296; doi:10.1371/journal.pone.0124844)
Supplement: S3 Table — (DOCX) [file pone.0124844.s003.docx]

**S3 Table. Univariate analysis of HFrEF vs. HFpEF.**

| **Metabolite** | **p-value** | **Mean(SD)** | | **HFrEF /  HFpEF** | **Fold Change** |
| --- | --- | --- | --- | --- | --- |
|  |  | **HFrEF** | **HFpEF** |  |  |
| Number of cases | - | 20 | 24 | - |  |
| BNP | 0.0117 | 367.5(306.25) | 154.62(159.64) | Up | 2.38 |
| NT.pro.BNP | 0.0282 | 238.43(294.38) | 110.05(139.64) | Up | 2.17 |
| C10 | 0.0089 | 0.24(0.19) | 0.31(0.13) | Down | -1.29 |
| C10.1 | 0.0732 | 0.23(0.07) | 0.28(0.09) | Down | -1.2 |
| C10.2 | 0.0426 | 0.05(0.02) | 0.07(0.03) | Down | -1.33 |
| C12 | 0.0060 | 0.1(0.05) | 0.15(0.07) | Down | -1.47 |
| C12.1 | 0.0791 | 0.23(0.06) | 0.29(0.1) | Down | -1.24 |
| C14 | 0.0657 | 0.05(0.01) | 0.05(0.01) | Down | -1.13 |
| C14.1 | 0.0391 | 0.15(0.06) | 0.18(0.06) | Down | -1.17 |
| C14.1.OH | 0.0200 | 0.02(0.01) | 0.02(0.01) | Down | -1.35 |
| C14.2 | 0.0790 | 0.03(0.02) | 0.04(0.03) | Down | -1.41 |
| C16 | 0.0562 | 0.12(0.04) | 0.14(0.04) | Down | -1.16 |
| C18.1 | 0.0267 | 0.16(0.06) | 0.2(0.08) | Down | -1.28 |
| C3.OH | 0.0542 | 0.06(0.01) | 0.07(0.01) | Down | -1.06 |
| C7.DC | 0.0083 | 0.05(0.01) | 0.07(0.03) | Down | -1.46 |
| C8 | 0.0070 | 0.23(0.15) | 0.26(0.08) | Down | -1.17 |
| PC.aa.C38.3 | 0.0919 | 63.04(24.87) | 54.02(19.15) | Up | 1.17 |
| PC.aa.C40.4 | 0.0121 | 4.23(1.21) | 3.45(1.39) | Up | 1.23 |
| PC.aa.C40.5 | 0.0162 | 13.96(4.37) | 11.44(4.62) | Up | 1.22 |
| PC.aa.C42.6 | 0.0504 | 0.78(0.17) | 0.71(0.17) | Up | 1.1 |
| PC.ae.C42.1 | 0.0464 | 0.37(0.08) | 0.32(0.09) | Up | 1.13 |
| PC.ae.C44.3 | 0.0562 | 0.12(0.02) | 0.11(0.02) | Up | 1.1 |
| lysoPC.a.C18.0 | 0.0609 | 24.62(5.96) | 21.6(5.06) | Up | 1.14 |
| SM.C16.0 | 0.0873 | 103.43(17.04) | 95.36(14.27) | Up | 1.08 |
| SM.C16.1 | 0.0732 | 17.02(4.28) | 15(3) | Up | 1.13 |
| SM.C20.2 | 0.0750 | 0.6(0.23) | 0.48(0.14) | Up | 1.24 |
| SM.C24.1 | 0.0173 | 50.49(8.39) | 45.07(8.14) | Up | 1.12 |
| X2.Hydroxybutyrate | 0.0001 | 4.14(10.18) | 35.6(27.78) | Down | -8.59 |
| X3.Hydroxybutyrate | 0.0216 | 29.61(44.27) | 69.71(61.32) | Down | -2.35 |
| Acetate | 0.0437 | 48.55(36.76) | 70.63(31.2) | Down | -1.45 |
