# Supplementary material for: Metabolomic Fingerprint of Heart Failure with Preserved Ejection Fraction
Source: PLoS One. 2015 May 26;10(5):e0124844. doi: 10.1371/journal.pone.0124844 (PMC4444296; doi:10.1371/journal.pone.0124844)
Supplement: S4 Table — (DOCX) [file pone.0124844.s004.docx]

**S4 Table. Logistic regression Analyses for the Models.**

| **Metabolite** | **B** | **S.E.** | **Z value** | **p-value** | **Change** |
| --- | --- | --- | --- | --- | --- |
| **HFpEF/Control** | | | | | |
| (Intercept) | -35.088 | 15.997 | -2.193 | 0.0283 | - |
| NT-proBNP | 0.997 | 0.324 | 3.077 | 0.0021 | Up |
| Octanoylcarnitine (C8) | 15.562 | 7.778 | 2.001 | 0.0454 | Up |
| Arginine | 6.002 | 2.941 | 2.041 | 0.0413 | Up |
| Sphingomyeline C20:2 | -9.226 | 3.479 | -2.652 | 0.0080 | Down |
| **HFrEF/Control** | | | | | |
| (Intercept) | -22.369 | 10.773 | -2.076 | 0.0379 | - |
| NT-proBNP | 6.351 | 3.011 | 2.110 | 0.0349 | Up |
| Acetoacetate | -0.643 | 0.351 | -1.830 | 0.0672 | Down |
| **HFrEF/HFpEF** | | | | | |
| (Intercept) | -69.054 | 33.610 | -2.055 | 0.0399 | - |
| BNP | 4.958 | 2.467 | 2.010 | 0.0445 | Up |
| Phosphatidylcholine diacyl C40:4 (PC aa C40:4) | 13.961 | 6.859 | 2.035 | 0.0418 | Up |
| Acetate | -0.483 | 0.267 | -1.807 | 0.0707 | Down |
| 2-Hydroxybutyrate | -0.368 | 0.196 | -1.881 | 0.0600 | Down |
| Pimelylcarnitine (C7.DC) | -9.621 | 5.092 | -1.889 | 0.0588 | Down |

HFpEF=Heart Failure with preserved Ejection Fraction, HFrEF=Heart Failure with reduced Ejection Fraction, BNP=B-type Natriuretic Peptide, NT-proBNP= N terminal proBNP
